# Hydrate of Chloral and Bromide of Potash Enemata in the Vomiting of Pregnancy

**Published:** 1879-09

**Authors:** 


Hydrate of Chloral and Bromide of Potash Enemata in the Vomiting of Pregnancy.—Recently having had a favorable result from hydrate of chloral by enema, given in a case of gastritis where vomiting had occurred almost incessantly for three weeks, we gladly give further publicity to the following note, in the *American Journal of Obstetrics and Diseases of Women and Children*, by D. B. Simons, M. D., Chief Surgeon to Ken Hospital, Yohohama. Japan :

I published in the *Medical Record* of May 15, 1874, the history of four cases of severe vomiting during the first month of pregnancy, as relieved by the administration of chloral hydrate by the rectum, in portions of from twenty to thirty grains, dissolved in gum water. I call the attention of the profession again to this method of treating these often very distressing cases, because I am more and more convinced of its great value, from repeated trials of it since. The Japanese physicians, whom I have instructed in its use, also report very favorably on it. In fact, they confidently inform me that it rarely fails. Since the first few cases in which I advised its use, I have learned that the bromide of potash, in equal proportions with the chloral, adds to its efficacy. I have also learned that in some cases the remedy must be pushed to a moderate degree of narcotism in order to secure the desired result. The amount of each portion of the drugs and their frequency of administration depends, therefore, on individual susceptibility to its influence, and must be prescribed accordingly. I also advised its use in obstinate vomiting from other causes. Following this suggestion, it was administered by one of my colleagues, Dr. Stewart Eldridge, in a case of vomiting from local peritonitis which had resisted all other modes of treatment. The result was most satisfactory, indeed, almost magical. I stated, in the article referred to, that I had nowhere seen the use of chloral for this particular purpose mentioned. Neither have I been able to find it since. I shall therefore claim to have first used and recommended it, till some prior claim is established.—*Canada Med. Record.*

